# Supplementary material for: Lung Spatial Profiling Reveals a T Cell Signature in COPD Patients with Fatal SARS-CoV-2 Infection
Source: Cells. 2022 Jun 7;11(12):1864. doi: 10.3390/cells11121864 (PMC9220844; doi:10.3390/cells11121864)
Supplement: Supplementary file 1 [file cells-11-01864-s001.zip › cells-1730691-supplementary.pdf]

Online Supplement

Lung spatial profiling reveals a T cell signature in the lungs of COPD patients with fatal SARS-CoV-2 infection

Chen Xi Yang[1,2], Michael Tomchaney[3], Manuel F. Landecho[4], Borja R. Zamacona[4], Marta Marin Oto[4], Javier Zulueta[5], Joshua Malo[6], Steve Knoper[6], Marco Contoli[7], Alberto Papi[7], Dragoş M. Vasilescu[1,8], Maor Sauler[9], Christof Straub[10], Cheryl Tan[10], Fernando D. Martinez[3], Deepta Bhattacharya[11], Ivan O. Rosas[12], Farrah Kheradmand[12], Tillie-Louise Hackett[1,2],*, Francesca Polverino[12,*]

*These authors contributed equally to the study

Methods

Study population

The study included one main cohort of patients who died of COVID-19 infection, and a secondary cohort of uninfected subjects. The main cohort included seven never-smokers and eleven ever-smokers with and without COPD who died from COVID-19 pneumonia following SARS-CoV2 infection at the University of Navarra Hospital, Pamplona, Spain. The secondary cohort included 19 subjects who did not have COVID-19 infection and underwent lung volume reduction surgery or transplant for the treatment of severe emphysema, or lung resection for a solitary peripheral nodule (the lung tissue studied was at least 10 cm away from the nodule) [1]. In this second cohort, none of the subjects studied had evidence of respiratory tract infection at the time of lung tissue sampling, and COPD was diagnosed according to the Global Initiative for Obstructive Lung Disease [2] recommendations.

All the study subjects defined as ever-smokers had a smoking history of at least 10 pack/years. All ever-smokers and COPD patients had ceased smoking for at least 1 year prior to the study.

Spatial gene profiling of parenchyma, bronchiole, and vessel tissues

The Nanostring GeoMX Digital Spatial Profiler (DSP) RNA slide preparation was followed according to the manufacturer's instruction (https://nanostring.com/products/geomx-digital-spatial-profiler/geomx-rna-assays). Briefly, the slides were baked, deparaffinized, and antigen retrieval was performed using Tris-EDTA pH 9.0. Next, samples were digested with proteinase K and hybridization was performed overnight using the Cancer Transcriptome Atlas and the COVID Immune Response Atlas RNA detection probes (https://www.nanostring.com/products/geomx-digital-spatial-profiler/geomx-rna-assays/geomx-cancer-transcriptome-atlas/). The same sections were then stained with fluorescently labeled SYTO13, pan-cytokeratin, and Cluster of Differentiation (CD)45 to identify tissue regions of interest (ROIs, **Figure 2A**). For each lung section, we randomly selected 16 total regions of interest (ROI) from the parenchyma, airways and vessels, uniformly spread throughout the section to ensure sample randomization. Each ROI was subdivided into compartments based on fluorescent cell-specific markers, and serial UV illumination of each compartment was used to sequentially collect the probe barcodes from the different cell types as described elsewhere. Once all of the indexing oligonucleotides were collected into a 96 well plate they were counted using next-generation sequencing. Each ROI was indexed using Illumina's i5xi7 dual-indexing system and the cDNA libraries were pair-end sequenced on the

NextSeq instrument (Illumina). Of the total of 1839 genes, 1803 were reliably measured across the dynamic range of gene expression panels (log2 normalized gene expression > 90th percentile of the expression of the negative control probes).

Spatial protein profiling of parenchyma, airways, and vessel structures

We used GeoMX Nanostring Digital Spatial Profiler to perform proteomic and transcriptomic analyses of the tissue sections from each subject. The samples were incubated with 41 oligo-labeled primary antibodies (See **Supplemental Table S2**): a Human Immune Cell Profiling Core, a Human Immune Activation Status Panel, a Human Immune Cell Typing Panel, a COVID-19 Immune Monitoring Panel, and custom-labelled antibodies against Syndecan-1, CD10, CD21 (all from abcam, Cambridge, MA). The slides were then loaded onto the GeoMX DSP and 16 ROIs were randomly selected per slide, and analyzed for protein expression. All the indexing oligonucleotides were collected into a 96 well plate and were then hybridized to fluorescent barcodes using GeoMX Hyb Codes. After hybridization, samples were processed using the using the nCounter system according to the manufacturer's instructions.

ACE2 and CD45RO immunofluorescence staining of FFPE lung tissue sections

The full staining protocol is described elsewhere [3]. Lung FFPE sections were deparaffinized, and antigen (Ag) retrieval was performed by treating the slides immersed in 0.01 M sodium citrate and 2 mM citrate buffer (pH 6.0) in a microwave. To identify ACE2- and CD45RO-positive cells within the alveolar and bronchiolar epithelium, lung sections were incubated with: 1) murine monoclonal antibody to CD45RO at 37 °C for 2 h (1:100, Abcam, Cambridge), followed by Alexa 488 conjugated goat anti-mouse IgG (H+L) at 37 °C for an hour (1:100, Fisher Scientific); or 2) rabbit polyclonal to ACE2 (1:100, Abcam) at 37 °C for an hour followed by Alexa-488 conjugated goat anti-rabbit IgG (H+L) at 37 °C for an hour (1:100, Fisher Scientific, Hampton, NH). Then, a goat polyclonal antibody to EPCAM was added overnight at 4 °C (1:100, Bio-Techne, Minneapolis, MN), followed by Alexa-568 conjugated rabbit anti-goat IgG (H+L) at 37 °C for an hour (1:100, Fisher Scientific).

To identify ACE2- and CD45RO- positive cells in the endothelial wall, lung sections were incubated at 37°C for 2 h with rabbit polyclonal antibody to ACE2 (1:50, abcam, ab15348), or murine monoclonal antibody to CD45RO (1:100, abcam,) at 37 °C for an hour, followed by Alexa-488-conjugated goat anti-rabbit IgG (H+L) or anti-mouse IgG (1:100, Fisher Scientific), respectively. Then, a sheep polyclonal to Von Willebrand Factor was applied for 2 h at 37 °C

(1:50, abcam), followed by Alexa 647 conjugated donkey anti-sheep IgG (H+L) at 37 °C for an hour (1:100, abcam). Lung sections were also immuno-stained with appropriate isotype-matched non-immune control antibodies.

For each sample, 20 randomly-selected high-magnification fields for each tissue type (alveoli, airways, and vessels) were evaluated using an Olympus epi-fluorescence microscope (Olympus Global Corporation). The area of the alveolar, bronchiolar basement membrane (BM), and vascular tissue measured was quantified using a Metamorph software (Molecular Devices, San Jose, CA) as we previously published[3,4]. The number of parenchymal, vascular, and bronchial $ACE2^+$ and $CD45RO^+$ cells were counted in separate analysis by two independent investigators blindly, and normalized by each tissue area. The data were expressed as either mean or median number of parenchymal, bronchial, and vascular $ACE2^+$ and $CD45RO^+$ cells/ alveolar, bronchial, and vascular tissue areas, respectively.

Statistics

The cellular proportions of the immune cell types between tissue types and disease conditions were inferred using the R package "SpatialDecon", a deconvolution method developed for the NanoString GeoMX RNA assay. The "Lung plus neutrophil" panel and the "Tcell_Adult_Lung_10x" panel were used to predict the cellular proportions of each ROI.

Tables

Table S1. Demographic and clinical characteristics of patients without COVID-19

	n	NS	ES	COPD	p
**Total Partecipants (n)**	19	4	6	9	
**Age**	19	63 [58-69]	71 [67, 76]	64 [62,68]	0.199
**Gender (M/F)**	19	2/2	5/1	7/2	0.319
**Smoking habit (current/former smoker)**	19	NA	0/6	0/9	NA
**FEV1 (% predicted)**	18	91% (16)	90% (18)	38% (24)	0.0004
**Comorbidities**					
Hypertension	19	3 (75%)	3 (50%)	3 (30%)	0.164
Other cardiovascluar diseases	19	2 (50%)	2 (30%)	4 (44%)	0.669
Diabetes Mellitus	19	2 (50%)	0	1 (11%)	0.052 (NS vs ES)
**Medications**					
Inhaled corticosteroids	18	0	0	6 (67%)	0.009
LABA/SABA/LAMA	18	0	0	8 (89%)	0.002
Oral corticosteroids	18	0	1 (17%)	3 (33%)	0.187
ACEi/ARB	18	2 (50%)	2 (30%)	2 (22%)	0.633

Data are expressed as the median [interquatile range] for continuous variables and n (%) for categorical variables. NS: never-smokers; ES: ever-smokers without COPD; LABA: long-acting beta-agonists; SABA: short-acting beta-agonists; LAMA: long-acting muscarinig agents; ACEi: ACE-inhibitors; ARB: angiotensin receptor blockers.

Table S2: Nanostring GeoMX Protein Targets

Protein	Code Class	Protein Group
**S6**	Control	Housekeepers;All targets
**Rb IgG**	Negative	Background;All targets
**Ki-67**	Endoge-nous	Proliferation;All targets
**CD45**	Endoge-nous	Total Immune;All targets
**PD-1**	Endoge-nous	T cells;Checkpoint;T cell Activation;All targets
**CD68**	Endoge-nous	M2 Macrophage;Myeloid;Macrophage;All targets
**GZMB**	Endoge-nous	T cell Activation;Cytotoxicity;All targets
**Ms IgG1**	Negative	Background;All targets
**GAPDH**	Control	Housekeepers;All targets
**Histone H3**	Control	Housekeepers;All targets
**CTLA4**	Endoge-nous	T cells;Checkpoint;T cell Activation;Th cells;All targets
**PD-L1**	Endoge-nous	Checkpoint;Myeloid Activation;All targets
**Fibronectin**	Endoge-nous	Stroma;Fibroblasts;All targets
**CD20**	Endoge-nous	B cells;All targets
**CD4**	Endoge-nous	T cells;Myeloid;Th cells;All targets
**CD8**	Endoge-nous	T cells;CD8 T cells;All targets
**Ms IgG2a**	Negative	Background;All targets
**HLA-DR**	Endoge-nous	Antigen Presentation;MHC2;All targets
**CD3**	Endoge-nous	T cells;All targets
**PanCk**	Endoge-nous	Tumor;Epithelial;All targets
**Beta-2-microglobulin**	Endoge-nous	Tumor;Antigen Presentation;All targets
**CD11c**	Endoge-nous	DC;Myeloid;All targets
**SMA**	Endoge-nous	Stroma;All targets
**CD56**	Endoge-nous	NK cells;All targets
**SARS-CoV-2 Spike**	Endoge-nous	Virus;All targets
**Cathepsin L/V/K/H**	Endoge-nous	Protease;All targets
**TMPRSS2**	Endoge-nous	Protease;All targets
**DDX5**	Endoge-nous	Immune Response;All targets

**ACE2**	Endoge-nous	Viral Receptor;All targets
**CD27**	Endoge-nous	T cells;T cell Activation;All targets
**CD80**	Endoge-nous	Myeloid;Myeloid Activation;All targets
**CD40**	Endoge-nous	Myeloid;Myeloid Activation;All targets
**CD44**	Endoge-nous	T cell Activation;All targets
**CD25**	Endoge-nous	T cells;T cell Activation;Tregs;All targets
**PD-L2**	Endoge-nous	Checkpoint;All targets
**CD127**	Endoge-nous	T cells;Naive and Memory;All targets
**ICOS**	Endoge-nous	T cell Activation;All targets
**CD14**	Endoge-nous	Myeloid;Monocyte;All targets
**CD45RO**	Endoge-nous	T cells;Memory;All targets
**FOXP3**	Endoge-nous	T cells;Th cells;Tregs;All targets
**CD34**	Endoge-nous	Hematopoietic;All targets
**FAP-alpha**	Endoge-nous	Stroma;Fibroblasts;All targets
**CD163**	Endoge-nous	M2 Macrophage;Myeloid;Macrophage;All targets
**CD66b**	Endoge-nous	Myeloid;Neutrophil;All targets
**CD21**	Endoge-nous	Custom Target;All targets
**Syndecan-1**	Endoge-nous	Custom Target;All targets
**CD10**	Endoge-nous	Custom Target;All targets

Figure Legends

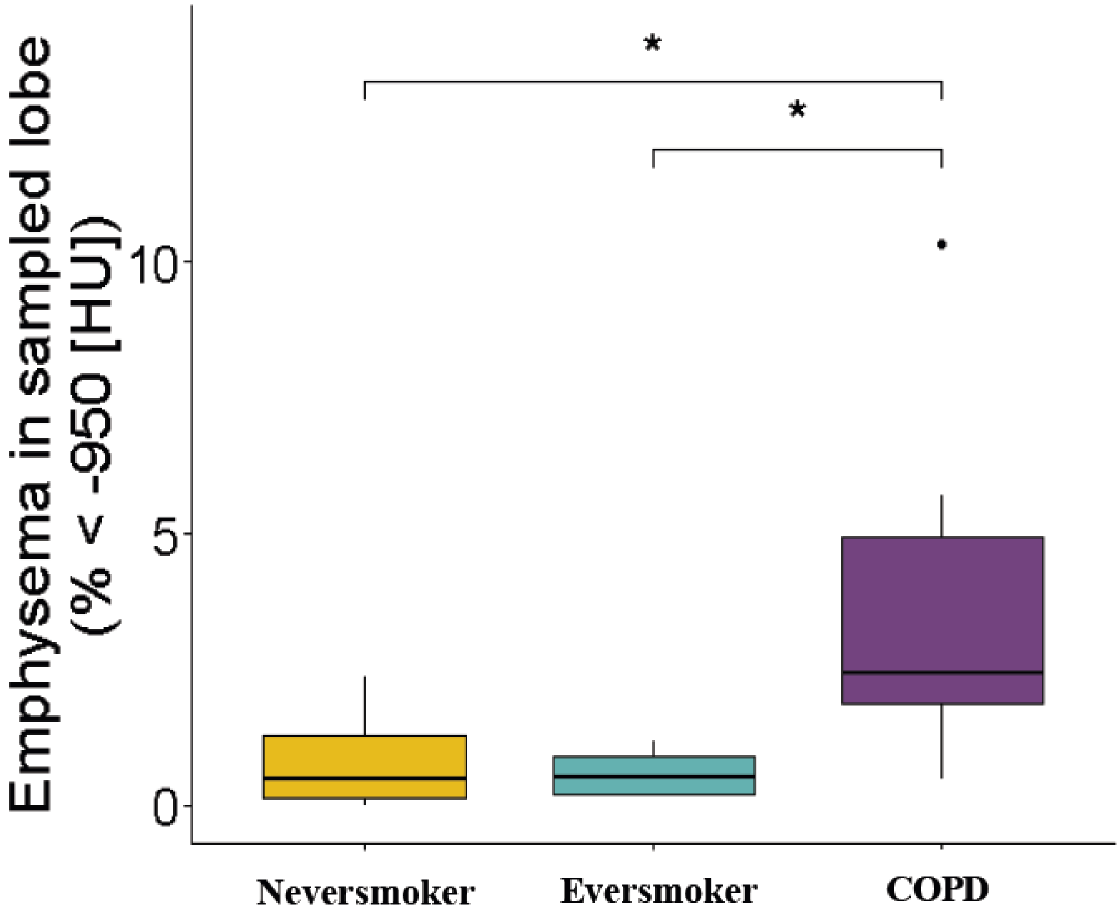

Figure S1. Emphysema measurements in the COVID-19 patient cohort **(A)** Thoracic computed tomography (CT) scans from never-smokers (NS), ever-smokers (ES), and patients with COPD within 4-10 days before death due to SARS-CoV-2 infection were assessed for emphysema (% lower attenuation area (LAA) <-950 Hounsfield units (HU), using Vida Vision software (Version 2.20, Iowa, USA)**.** COPD patients had a greater percentage of emphysema (%LAA_{950}) compared to never-smoker and ever-smokers. * indicates, $P < 0.05$.

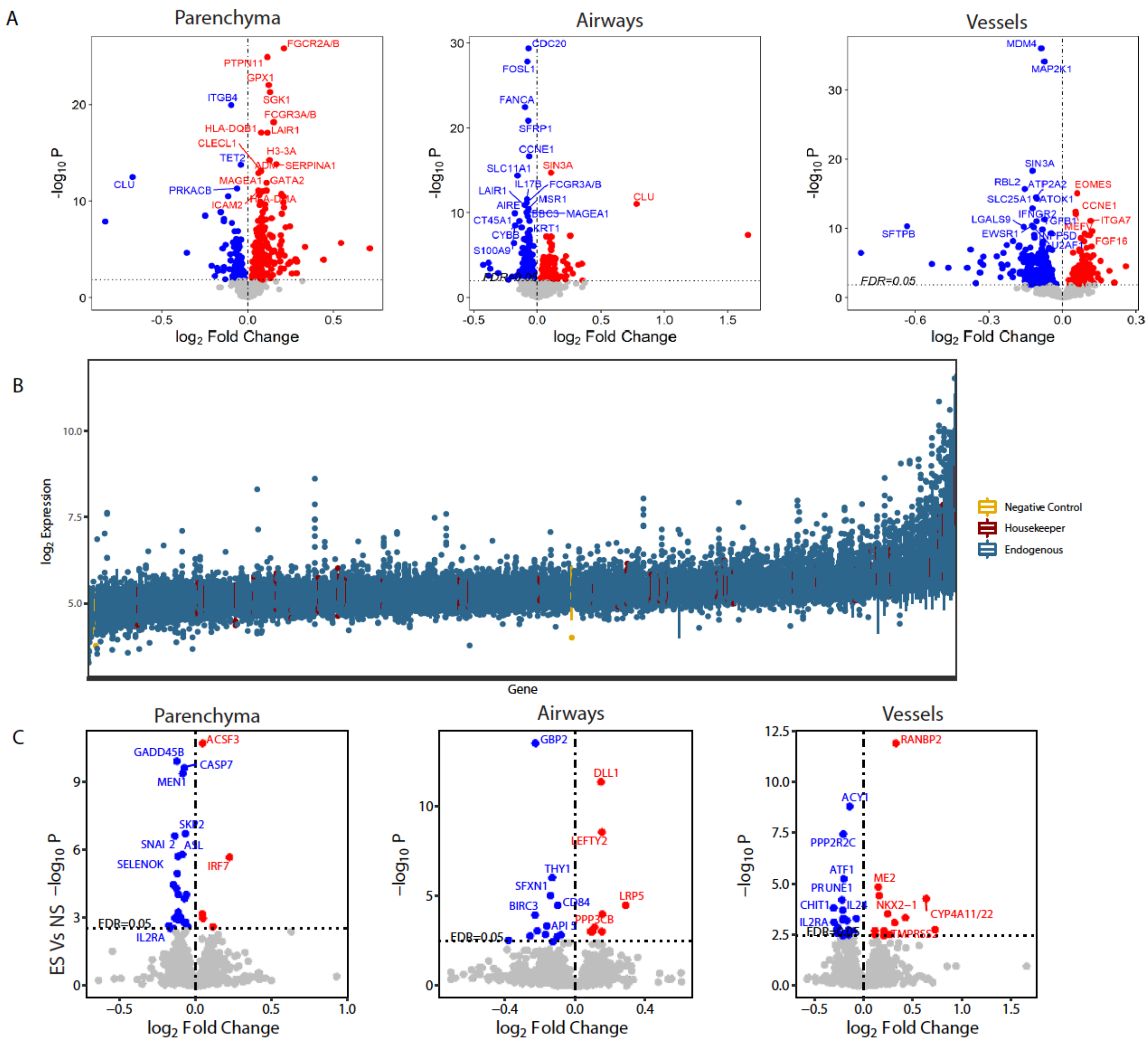

Figure S2. Gene signatures of tissue structures within all the COVID-19 patient cohort (**A**) Volcano plots showing the genes expressed specifically in the parenchyma, airways, and vessels of all patients who died of COVID-19. (**B**) Box-plots showing the dynamic range of all genes assessed using the NanoString GeoMX DSP panel. (**C**) Volcano plots showing the differential gene expression in the parenchyma, airways, and vessels of never- (NS) vs ever-smokers (ES) controls. For all volcano plots, the data were assessed using a generalized estimating equations (GEE) accounting for multiple ROIs per case and was adjusted for age. The red dots indicate up-regulated genes and the blue dots down-regulated genes in COPD patients versus never- and ever-smokers for the specific tissue structures. The horizontal dotted line indicates the significance threshold of FDR < 0.05, and the vertical dashed line indicates the effect size of 0. The grey dots indicate genes that were not significant at FDR < 0.05. The top genes ranked by *p*-value are labelled with their gene symbols.

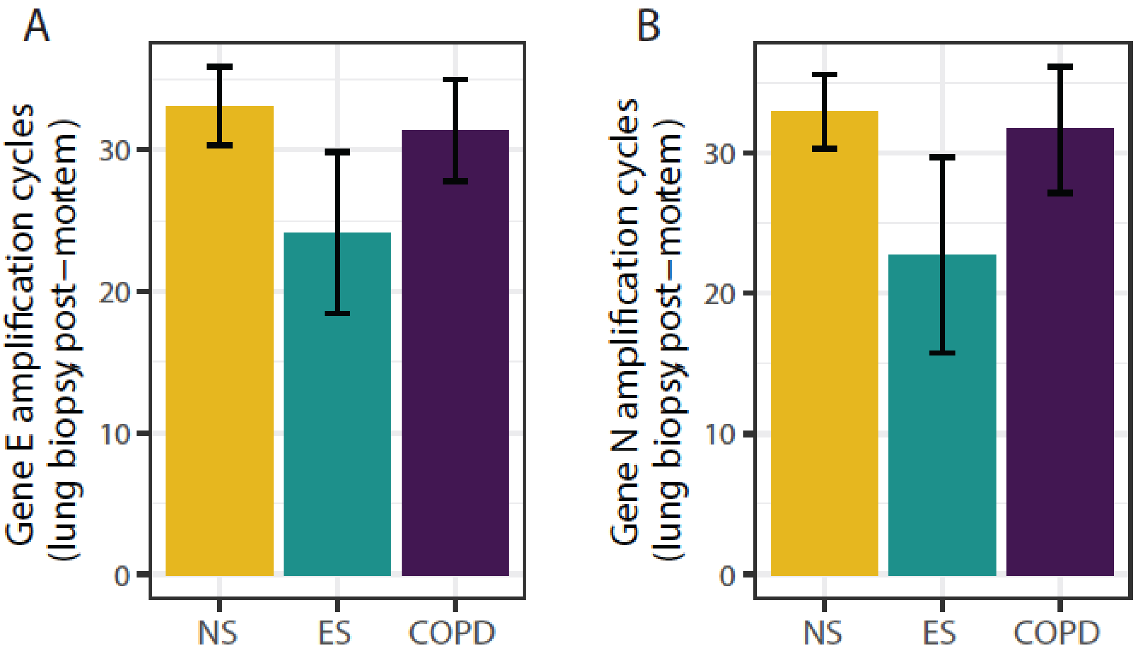

Figure S3: Envelope (*E*) and nucleocapsid (*N*) gene expression levels in lungs of all patients who died of COVID-19 (**A**) *Gene E (envelope)* and (**B**) *N (nucleocapside)* RNA expression levels were measured in lungs from never- (NS) and ever- (ES) smoker controls and COPD patients who all died of SARS-CoV-2 infection using rt-PCR.

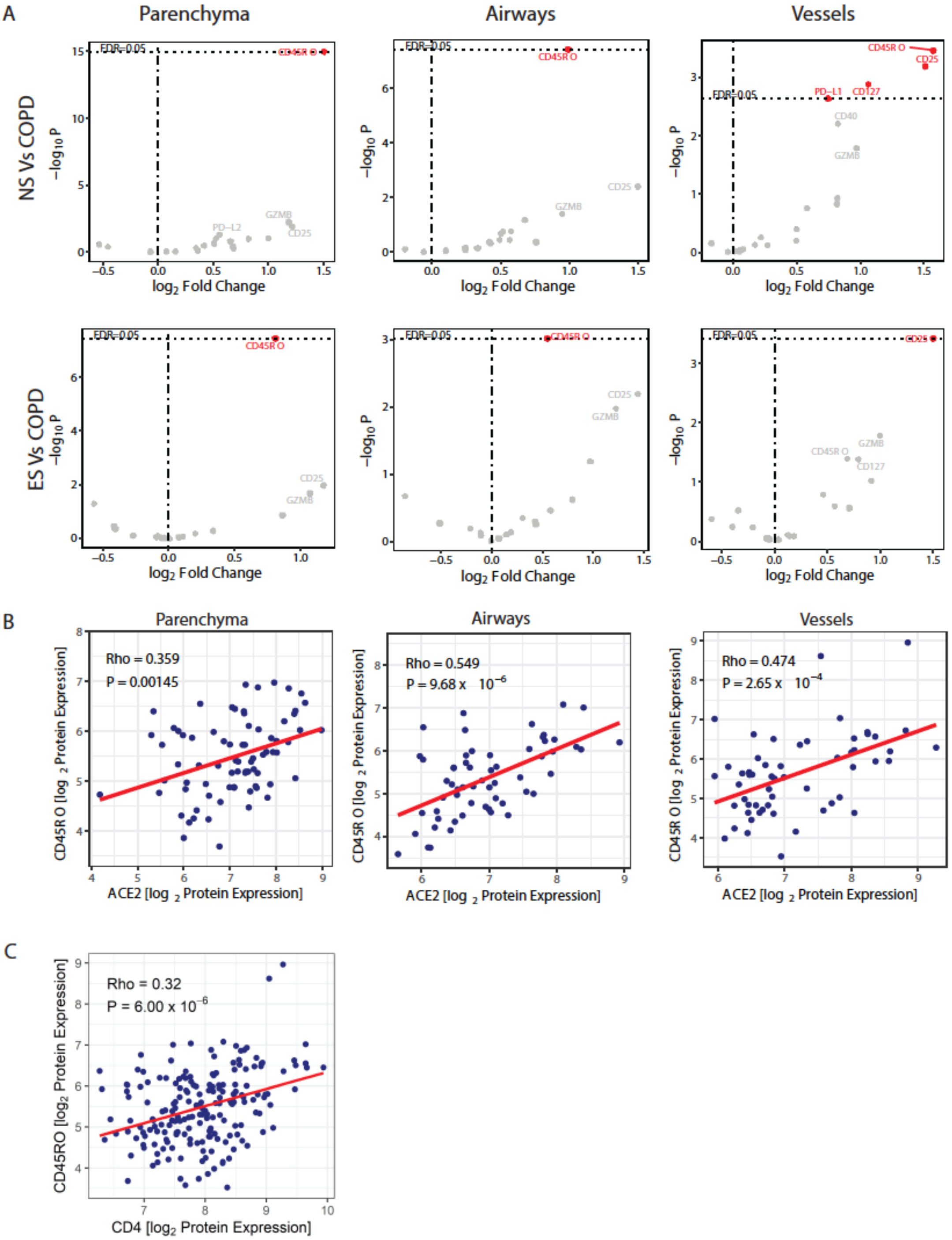

Figure S4. T cell protein signatures within all the COVID-19 patient cohort, and correlations between CD45RO and ACE2 protein expression (**A**) Volcano plots showing the protein markers assessed in the parenchyma, airways, and vessels of all patients who died of COVID-19. The data were assessed using a generalized estimating equations (GEE) accounting for multiple ROIs per case, and was adjusted for age. The red dots indicate up-regulated genes and the blue dots down-regulated genes in COPD patients versus never- and ever-smokers for the specific tissue structures. The horizontal dotted line indicates the significance threshold of FDR < 0.05, and the vertical dashed line indicates the effect size of 0. The grey dots indicate genes that were not significant at FDR < 0.05. The top proteins ranked by p-value

are labelled with their gene symbols. (**B**) Correlations between CD45RO protein expression and ACE2 protein expression in parenchyma, airways and vessels. The Rho and P values are given, $p < 0.05$ was considered significant. (**C**) Correlations between CD45RO protein expression and CD4 protein expression in all the tissue type studied. The Rho and P values are given, $p < 0.05$ was considered significant.

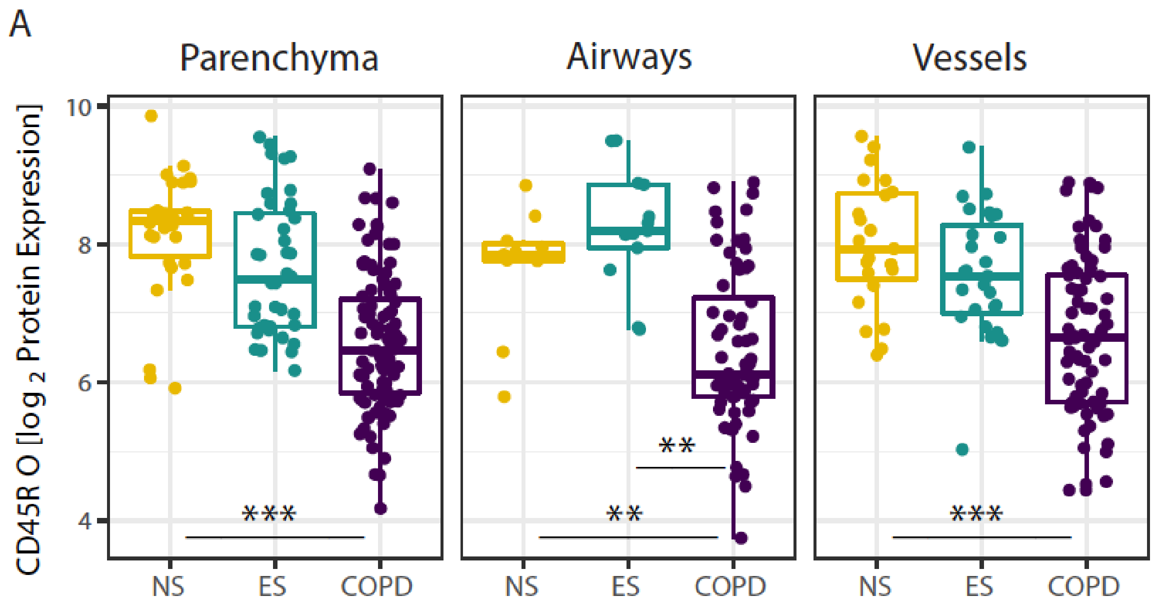

Figure S5. CD45RO protein expression in patients without COVID-19 (**A**) CD45RO protein expression levels were quantified in the parenchyma, airways, and vessel regions of interest (ROIs) in lungs from patients with COPD versus ever- (ES) and never-smoker (NS) controls without SARs-CoV-2 infection, using GeoMX digital spatial profiling. The data were assessed using a generalized estimating equations (GEE) accounting for multiple ROIs per case and was adjusted for age. The box plots represent the median and interquartile range, the error bars are the 5th and 95th percentile, and each dot represents a single ROI. * indicates $P < 0.05$, and *** indicates $P < 0.001$.

References

1. Sullivan JL, Bagevalu B, Glass C, et al. B Cell Adaptive Immune Profile in Emphysema-Predominant COPD. *Am J Respir Crit Care Med*. Dec 1 2019;200(11):1434-1439. doi:10.1164/rccm.201903-0632LE
2. Mirza S, Clay RD, Koslow MA, Scanlon PD. COPD Guidelines: A Review of the 2018 GOLD Report. *Mayo Clin Proc*. Oct 2018;93(10):1488-1502. doi:10.1016/j.mayocp.2018.05.026
3. Polverino F, Wu TD, Rojas-Quintero J, et al. Metformin: Experimental and Clinical Evidence for a Potential Role in Emphysema Treatment. *Am J Respir Crit Care Med*. May 25 2021;doi:10.1164/rccm.202012-4510OC
4. Polverino F, Cosio BG, Pons J, et al. B Cell-Activating Factor. An Orchestrator of Lymphoid Follicles in Severe Chronic Obstructive Pulmonary Disease. *Am J Respir Crit Care Med*. 9/15/2015 2015;192(6):695-705. Not in File. doi:10.1164/rccm.201501-0107OC [doi]
